# Research on pore characteristics and macroscopic performance quantitative prediction of desert sand recycled aggregate concrete based on NMR and grey theory

**DOI:** 10.1371/journal.pone.0349560

**Published:** 2026-06-03

**Authors:** Xiumei Zheng, Wenbang Zhu, Yali Cao, Xinjie Wang, Enze Hao, Dali Zhang, Wentao Chu, Baochen Tang

**Affiliations:** 1 College of Civil Engineering, Kashi University, Kashi, China; 2 Xinjiang Key Laboratory of Engineering Materials and Structural Safety, Kashi University, Kashi, China; 3 School of Architectural Engineering, Changji Vocational and Technical College, Changji, China; Guizhou University, CHINA

## Abstract

Excessive extraction of river sand and the accumulation of construction waste severely restrict the green development of civil engineering. The resource utilization of desert sand (DS) and recycled aggregates (RA) is of great significance for the sustainable construction in arid desert areas of Xinjiang. To reveal the mechanism of synergistic regulation of concrete properties by the combined control of calcined mica hydroxide (CLDHs), desert sand and recycled aggregates, and to establish a quantitative correlation between microstructure and macroscopic properties, this paper uses the CLDHs dosage, DS substitution rate, and RA substitution rate as test parameters to prepare desert sand recycled aggregate concrete (DSRAC). Through testing methods such as compressive strength, rapid chloride ion migration, nuclear magnetic resonance (NMR), scanning electron microscopy (SEM), and X-ray diffraction (XRD), the macroscopic properties and microstructural evolution laws of the materials are systematically analyzed, and a quantitative performance prediction system is constructed based on grey entropy correlation analysis and GM (1,4) model. The results show that CLDHs, DS, and RA achieve synergistic optimization of pore structure through physical filling, ionic curing, and secondary hydration; when the CLDHs dosage is 6%, the DS and RA substitution rates are both 30%, the comprehensive performance of the concrete is the best, and the 28 d compressive strength is basically the same as the control group, with a 33.09% reduction in chloride ion diffusion coefficient. The internal pores of DSRAC are mainly gel pores, and the total porosity and transitional pore ratio have a significant impact on the macroscopic properties, with a correlation coefficient greater than 0.8. The GM (1,4) prediction model established based on key pore structure parameters has a relative error of less than 8% and high accuracy. The research results can provide theoretical basis and technical support for the preparation, performance evaluation, and engineering application of green recycled concrete in arid saline areas.

## 1. Introduction

Natural aggregates have been over-mined for a long time, leading to increasingly prominent shortages of resources and ecological pressure. Seeking green and sustainable alternatives to aggregates has become an important development direction in the field of civil engineering [1–3]. The combined application of desert sand and recycled aggregates in concrete production can not only effectively alleviate the shortage of natural aggregates, but also promote the resource utilization of solid waste and the efficient development of desert resources, which is of great significance for building a green, low-carbon, and sustainable building material system [4–6].

The macroscopic performance of concrete is intimately associated with its pore microstructure. The type and content of aggregates play a critical role in modifying pore structure characteristics and the cement hydration kinetics, which in turn exert a direct effect on the mechanical behavior and durability performance of concrete [7–10]. In recent decades, researchers globally have undertaken extensive investigations into recycled aggregate concrete, fiber-reinforced concrete, and green low-carbon concrete, delivering notable progress in material manufacturing, performance tuning, and engineering deployment [11–14]. Yang et al. [15] revealed that DS can accelerate the early hydration kinetics of cement via the effects of particle dispersion, pore filling, and nucleation, resulting in an enhancement of the early-age strength of RAC. When exposed to coupled sulfate attack and freeze-thaw cycles, a 50% DS replacement rate can effectively restrain the expansion of surface damage in concrete, diminish the proportion of gel pores, and strengthen the sulfate resistance of concrete. Liu et al. [16] revealed that the hardened mortar attached to the RA surface can induce a filling effect and experience secondary hydration, resulting in the formation of hydration products that densify the pore structure and, to a certain extent, refine the defects within the interfacial transition zone (ITZ). However, RA also has inherent limitations. The residual hardened mortar adhering to the RA surface demonstrates pronounced water absorption, which disturbs the water transport behavior in the ITZ Furthermore, the calcium hydroxide (CH) contained within this residual mortar reacts with water and silica during the mixing stage, yielding C-S-H hydration products that tend to induce an uneven matrix microstructure in the ITZ. If the cement hydration reaction is insufficient, it will also weaken the microstructure density of recycled aggregate concrete [17,18]. Zhu et al. [19] demonstrated that the ultrafine particle characteristics of DS can exert a physical filling effect and form a stable bond with the hydration products, helping to enhance the concrete density. Luo et al. [20] revealed that the moderate addition of desert sand can optimize the aggregate skeleton gradation and densify the pore structure, resulting in an improvement in the compactness of concrete. Furthermore, the active SiO_2_ in desert sand can promote the pozzolanic reaction to generate C-S-H hydration gel, which enhances the ITZ bonding and the mechanical strength of the material. However, existing studies mostly focus on the influence of DS or RA substitution alone. Regarding the synergistic mechanism of their combined substitution and the regulation of DSRAC performance by CLDHs, especially the research on the multi-component dosage-dependent action law, is still not systematic, and there is a lack of quantitative correlation analysis between microstructure and macroscopic properties, making it difficult to provide precise guidance for material ratio optimization.

To accurately assess the comprehensive performance of DSRAC, it is crucial to establish a quantitative correlation between micro and macro performance. Currently, the evaluation of concrete performance mostly relies on macroscopic test data, while the pore structure, as the key link connecting micro characteristics and macro performance, has not been fully quantified with respect to its intrinsic correlation with compressive strength and chloride ion permeation performance [21–24]. Grey entropy correlation analysis, as an efficient multi-factor quantitative analysis method, has the advantages of requiring few samples and reliable results, and has been widely applied to reveal the strength of the correlation between concrete performance and influencing factors [25–27]; the grey prediction model, with its low data requirements and convenient calculation, demonstrates significant advantages in the field of concrete performance prediction, especially suitable for quantitative analysis in systems with incomplete information [28–30]. Zhang et al. [25] found through grey entropy correlation analysis that the T_2_ spectral area and large porosity are key factors significantly affecting the mechanical properties of self-compacting concrete. Moreover, the GM (1,3) model built upon.NMR. experimental data can effectively predict the mechanical behavior of this type of concrete. Dong et al. [26] confirmed through grey entropy correlation analysis that the correlation between compressive strength of concrete and the saturation degree of binding liquid, harmless porosity rate, and secondary harmful porosity rate is all higher than 0.8, indicating a significant correlation between pore structure characteristics and mechanical performance; and based on the nuclear magnetic resonance pore structure parameters, a GM (1,4) strength prediction model was constructed. Zhu et al. [31] revealed the mechanism and evolution law of performance deterioration of desert sand semi-flexible pavement grouting material under long-term sulfate erosion, and achieved high-precision life prediction using the GM (1,1) model, providing an effective method for the evaluation of concrete durability. Relevant studies have shown that the application of grey theory and GM models provides a feasible path for establishing the correlation between macroscopic performance of concrete and microstructure. However, the application of these methods in the DS, RA, and CLDHs composite system is still relatively limited, and the prediction accuracy and applicability of the macroscopic performance of DSRAC still need to be verified.

This study, based on the special environment of the desert-salt soil in southern Xinjiang, with CLDHs dosage, desert sand replacement rate and recycled aggregate replacement rate as key parameters, systematically investigated the macroscopic mechanical properties, durability performance and microstructure evolution laws of DSRAC under the combined effect of multiple components. Through grey entropy correlation analysis and GM (1,4) model, a quantitative prediction relationship between pore structure and macroscopic properties was established. The research focused on green low-carbon, high-performance and engineering practicality. It clarified the cooperative regulation mechanism and technical advantages of the composite system, and could provide theoretical basis and technical support for the engineering application of green recycled concrete in desert-salt areas.

## 2. Materials and testing methods

### 2.1 Raw materials

**Fig 1:**
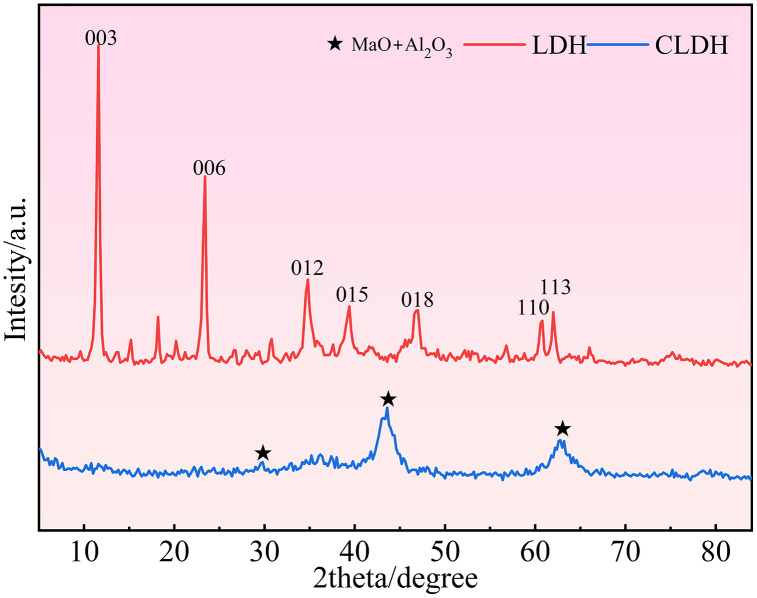
XRD spectra of CLDH before and after sintering.

**Fig 2:**
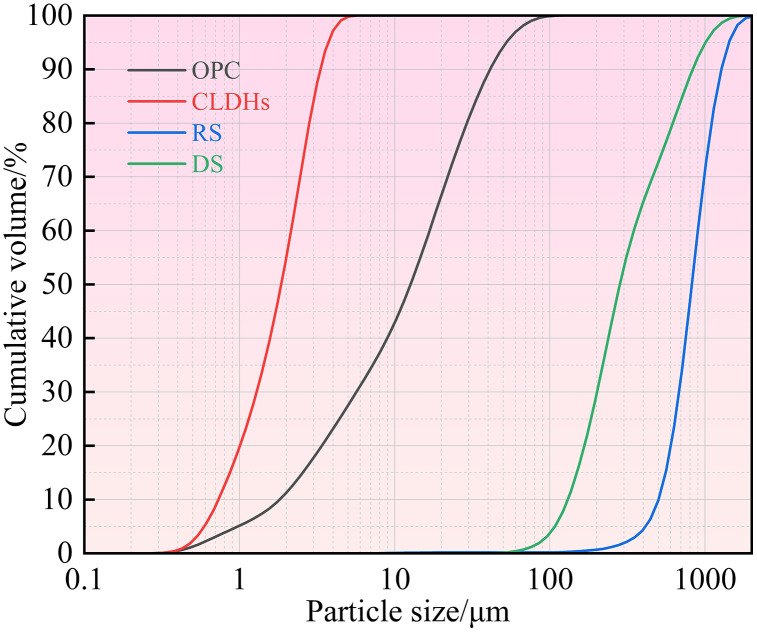
Particle size distribution of OPC, CLDHs, RS and DS.

**Fig 3:**
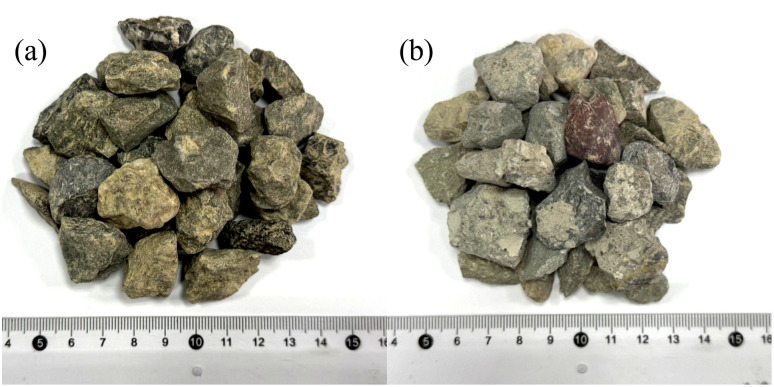
Coarse aggregate. **(a)** Crushed stone, **(b)** Recycled aggregate.

For this experiment, P·O 42.5 grade ordinary silicate cement produced by Kashi Tianshan Cement Factory was selected as the cementitious material, and hydrotalcite was used as the supplementary cementitious material. Hydrotalcite is a type of anionic nano-mineral material with a layered crystal structure, which inherently possesses excellent ion adsorption properties. To further enhance its adsorption activity, hydrotalcite was subjected to 5 h high-temperature calcination treatment at 500 °C. During calcination, the elimination of interlayer crystalline water and gaseous components leads to the generation of numerous micropores inside the material, which in turn elevates its specific surface area and exposes additional active adsorption sites. The XRD spectra of hydrotalcite before and after calcination are shown in Fig 1. The main chemical compositions of cement and calcined hydrotalcite are presented in Table 1. Fine aggregates were selected as Ⅱ grade river sand (RS) that met the requirements of GB/T 14684−2022, as well as DS taken from the surrounding areas of Wupar Town, Shifu County. The particle size distribution and specific surface area of the washed and sieved RS and DS were tested according to the above standards, and the results are shown in Table 2. The particle size distribution curves of cement, calcined hydrotalcite, RS, and DS are shown in Fig 2. RA were obtained by crushing construction waste concrete with a jaw crusher and grading with standard screens. Their particle size distribution was basically consistent with that of natural crushed stones. The physical properties of RA were tested in accordance with GB/T 14685−2022, and the content of old mortar attached to RA was determined according to GB/T 25177−2010. Fig 3 presents the macroscopic appearance of natural aggregates and RA, and Table 3 summarizes the critical physical performance parameters. A conventional polycarboxylate superplasticizer, supplied by Sichuan Dongrun Baisheng New Materials Co., Ltd. with a water reduction rate above 25%, was adopted as the water-reducing admixture.

**Table 1:** Oxide chemical compositions of OPC and CLDHs (wt.%).

Materials	SiO_2_	Al_2_O_3_	CaO	Fe_2_O_3_	MgO	K_2_O	SO_3_
OPC	25.4	7.1	55.6	4.9	1.3	1.4	3.3
CLDHs	1.1	40.9	2.7	0.1	53.8	0.01	1.0

**Table 2:** Particle size distribution characteristics and specific surface area properties of RS and DS.

Particle size/mm	River sand			Desert sand		
	Grader retained percentage/%	Cumulative retained percentage/%	Specific surface area/m^2^·kg ^−1^	Grader retained percentage/%	Cumulative retained percentage/%	Specific surface area/m^2^·kg ^−1^
2	0.08	0.08	3.372	0.00	0.00	9.073
1	27.38	27.46		7.71	7.71	
0.5	62.67	90.13		27.02	34.73	
0.25	8.33	98.46		48.98	83.71	
0.075	1.54	100.00		16.29	100.00	

**Table 3:** Fundamental physical characteristics of coarse aggregates.

Coarse aggregate	Continuous gradation/ mm	Dry density/ g·cm^-3^	Water Absorption/ %	Crushing value/%	Old mortar content/%
Crushed stone	5–25	2.65	0.45	12.5	/
Recycled aggregate	5–25	2.26	3.2	19.2	14.7

### 2.2 Mix proportion design and sample preparation

The concrete mixture proportions were determined in accordance with the provisions of Chinese national standard JGJ 55–2012 [32]. The water-cement ratio was fixed at 0.4. The test adopted the internal admixture method, replacing the cement with 1%, 3%, and 6% of calcined talc respectively; at the same time, 10%, 20%, and 30% of desert sand was used to replace the river sand in equal amounts, and the natural crushed stone was replaced with recycled coarse aggregates in the same proportion. Thus, DSRAC was prepared. The detailed mix ratio parameters of each group of concrete are shown in Table 4.

**Table 4:** Design of mix ratio for DSRAC.

Specimen NO.	CLDHs/%	DS/%	Recycled aggregate/%	Water-binder ratio	water reducer/%	Consumption of test materials/kg·m^-3^							
						OPC	CLDHs	Water	River sand	Desert sand	Crushed stone	Recycled aggregate	water reducer
D0R0	0	0	0	0.4	1.5	420	0	168	580	0	1320	0	6.3
D0R10	1	0	10	0.4	1.5	415.8	4.2	168	580	0	1188	132	6.3
D0R20	1	0	20	0.4	1.5	415.8	4.2	168	580	0	1056	264	6.3
D0R30	1	0	30	0.4	1.5	415.8	4.2	168	580	0	924	396	6.3
D10R0	3	10	0	0.4	1.5	407.4	12.6	168	522	58	1320	0	6.3
D20R0	3	20	0	0.4	1.5	407.4	12.6	168	464	116	1320	0	6.3
D30R0	3	30	0	0.4	1.5	407.4	12.6	168	406	174	1320	0	6.3
D10R10	6	10	10	0.4	1.5	394.8	25.2	168	522	58	1188	132	6.3
D20R20	6	20	20	0.4	1.5	394.8	25.2	168	464	116	1056	264	6.3
D30R30	6	30	30	0.4	1.5	394.8	25.2	168	406	174	924	396	6.3

A detailed description of the DSRAC preparation procedure is provided below: Raw materials were proportioned as per the predetermined mix design, followed by homogeneous dry blending of cement, calcined hydrotalcite, fine aggregate, and coarse aggregate. The pre-dissolved water-soluble retarder was incorporated into the mixture, and wet mixing was executed for 120 s. Each DSRAC batch was cast into test molds with dimensions of Φ100 mm × 50 mm, 100 mm × 100 mm × 100 mm, and 40 mm × 40 mm × 40 mm, respectively, and consolidated via vibration on a shaking table. The freshly cast specimens were initially film-covered and cured under ambient laboratory conditions (20 ± 5 °C, relative humidity ≥ 50%) for 24 h prior to demolding, and subsequently transferred to a standard curing chamber maintained at 20 ± 2 °C and ≥ 95% relative humidity for continuous curing.

### 2.3 Test methods

#### 2.3.1 Mechanical property tests.

Pursuant to Chinese national standard GB/T 50081−2019 [33], the mechanical performance of DSRAC specimens at curing ages of 3 d, 7 d, and 28 d was experimentally investigated. Compressive strength measurements were carried out on 100 mm cubic specimens, with three replicate specimens assigned to each group. A hydraulic testing machine with a maximum load capacity of 2000 kN was utilized, and a loading rate of 0.5 MPa/s was imposed for all tests. The test was concluded upon failure of the specimen, at which time the peak load was recorded. The calculation formula for compressive strength is detailed in Eq. (1).


$$\begin{array}{c} R_{c}=\frac{F_{c}}{A} \end{array}$$ (1)

In the equation, *Fc* represents the failure load of the DSRAC (N), and *A* is the bearing area of the DSRAC in the test (mm²).

#### 2.3.2 Rapid chloride permeability test.

The chloride ion permeability of DSRAC at 28 d curing was determined using the Rapid Chloride Permeability Test (RCPT) method, strictly in accordance with GB/T 50082−2024 [34]. Cylindrical specimens (100 mm diameter × 50 mm height) were selected for testing. To eliminate permeation interference, the side surfaces of the specimens were first sealed with epoxy resin. The samples were subsequently placed in a vacuum saturation setup, where the vacuum pressure was adjusted to ≤ 133 Pa and sustained for 3 h. Deionized water was subsequently added to submerge the specimens completely under vacuum. Following vacuum release, the specimens were soaked at normal pressure for 18 ± 2 h to attain a saturated state. Prior to testing, the specimens were stored under high humidity conditions (RH ≥ 95%) and must be installed in the test chamber within 30 min to avoid inaccuracies caused by water evaporation. For the RCPT measurement, 0.3 mol/L NaOH solution was introduced into the anode chamber, and 3.0% NaCl solution was introduced into the cathode chamber. A constant DC voltage of 60 V was applied for 6 hours. The current traversing each specimen was recorded using a DTL-9T measuring instrument, and the total charge passed was calculated utilizing Eq. (2).


$$\begin{array}{c} Q=900\left(I_{\text{0}}+2I_{30}+2I_{60}+\cdots 2I_{330}+I_{360}\right) \end{array}$$ (2)

In the formula: *Q* represents the total electric flux (C); *I*_*0*_ is the initial current (A); It is the current at time t (A).

The electric flux must be normalized by using the reference values obtained from a standard sample with a diameter of 95 mm for the calculated Q. This can be converted according to Eq. (3).


$$\begin{array}{c} Q_{s}=Q_{x}{\left(95/x\right)}^{\text{2}} \end{array}$$ (3)

Here, *Qs* represents the electrical flux of the standard 95 mm sample (C); *Q*_*x*_ indicates the electrical flux of the non-standard sample (C), with *x* representing its diameter (mm). The calculations of the electrical flux and the correlation with the chloride ion diffusion coefficient are all based on the RCPT test conditions of complete saturation, standard curing for 28d, test temperature ranging from 20 to 25 °C, and constant voltage of 60 V for 6 h.

#### 2.3.3 Pore structure test.

A MesoMR12-060H-I multifunctional NMR analyzer, manufactured by Suzhou Niumai Analytical Instrument Co., Ltd., was employed to perform quantitative characterization of the micro-pore structure of DSRAC specimens with a molded dimension of 40 mm × 40 mm × 40 mm. The instrument exhibits an effective pore diameter detection range of 0.00002–200 μm. In the test pretreatment stage, specimens that had undergone standard curing for 28 d were placed in a vacuum water-retention device for 4 h of air extraction to completely remove air from the pores. Subsequently, the specimens were immersed in distilled water for 24 h of curing to ensure they achieved a fully saturated state. During the testing stage, core microstructural parameters of the specimens—including porosity, transverse relaxation time (T_2_) spectral distribution, and pore distribution characteristics—were accurately acquired using NMR technology. Based on the fundamental detection principle of NMR, the correlation between pore relaxation time and pore diameter can be expressed as Eq. (4):


$$\begin{array}{c} \frac{\text{1}}{T_{\text{2}}}=\rho_{\text{2}}\frac{S}{V} \end{array}$$ (4)

In the formula: S represents the specific surface area of the pores (cm²), V represents the pore volume (cm³), and *ρ*_*2*_ represents the surface relaxation intensity (μm/ms). In this study, the surface relaxation rate ρ_2_ of the cement-based concrete material is set at 5 μm/ms. This value is a generally accepted standard in the field of nuclear magnetic resonance pore structure analysis of concrete and has been widely used in similar studies to verify its rationality, ensuring the reliability of the pore size calculation and pore structure distribution results.

#### 2.3.4 SEM tests.

The microstructural characteristics of the DSRAC interior were examined using a Phenom Pro X field-emission scanning electron microscope. The sample preparation protocol is detailed below: Following the 28 d compressive strength testing, small specimens were cored from the DSRAC cubic samples, then sectioned and ground to produce rectangular specimens with dimensions of 5 mm × 5 mm × 2 mm. The specimens were then immersed in anhydrous ethanol for 24 h to terminate the hydration reaction, followed by vacuum drying treatment. Finally, the surfaces of the dried specimens were gold-sputtered to improve their electrical conductivity and eliminate charging effects during electron beam irradiation.

#### 2.3.5 XRD tests.

A TD-3500 X-ray diffractometer was employed to analyze the hydration products of DSRAC. The sample preparation workflow was conducted as follows: After completing the 28-day compressive strength tests, small samples were retrieved from the interior of the DSRAC cubic specimens. These samples were soaked in anhydrous ethanol for 24 h to stop the hydration reaction, then dried at 40 °C for 24 h, ground into powder, and sieved before XRD measurement. The XRD patterns were collected in the 2θ range of 5° to 85° with a scanning rate of 0.1°/s.

**Fig 4:**
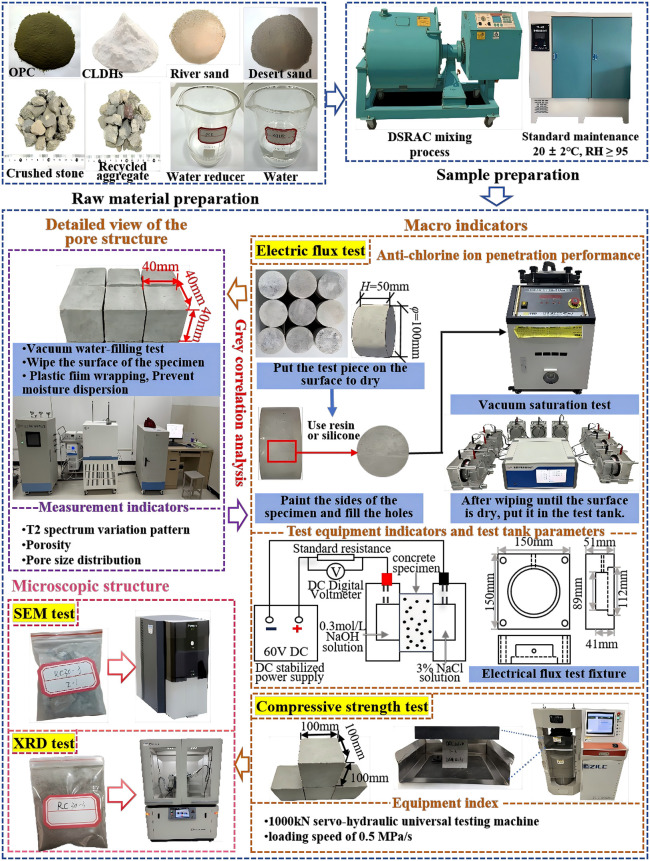
Setup and flow chart of multiple tests.

The test parameter settings and specific operation steps followed during the implementation of the above-mentioned experiment are shown in Fig 4.

## 3. Results and discussion

### 3.1 The influence of DS, RA and CLDHs on the compressive strength of DSRAC

**Fig 5:**
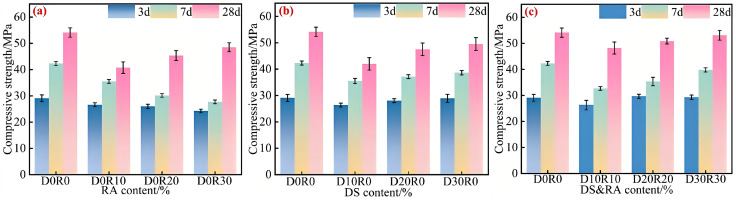
Different types of concrete compressive strength. **(a)** Effect of RA replacement ratio, **(b)** Effect of DS replacement ratio, **(c)** Synergistic effect of DS&RA replacement ratios.

Fig 5 summarizes the effect of different mix ratios on the compressive strength of concrete. At the 3 d curing stage, concrete specimens with mix ratios of D0R30, D30R0, and D30R30 exhibit compressive strengths of 24.26 MPa, 28.03 MPa, and 29.26 MPa, respectively. Relative to the control group D0R0, D0R30 and D30R0 experience strength losses of 16.57% and 1.6%, whereas D30R30 achieves a slight strength gain of 0.62%. At the 28 d curing age, the compressive strengths for the aforementioned three mixtures are determined to be 48.5 MPa, 49.5 MPa, and 53.1 MPa. When benchmarked against the control group D0R0, the corresponding strength reduction rates for D0R30, D30R0, and D30R30 are 10.40%, 8.55%, and 1.90%, respectively.

As can be seen from Fig 5(a), the 3 d compressive strength of concrete shows no obvious change when the RA substitution rate is increased from 10% to 30%. This demonstrates that the dominant reason for the strength degradation in this group is the reduction in the proportion of the cementitious system after 1% CLDHs substitution for cement, which subsequently results in a diminished amount of hydration products. The results in Fig 5(b) indicate that, with 3% CLDHs replacing cement, a rise in DS dosage from 10% to 30% results in a 9.59% improvement in the 3 d compressive strength of the concrete. This strength enhancement is attributable to the dual functionality of DS within the cement matrix, serving as both a dispersant and a filler: its ultrafine particles not only furnish additional nucleation sites for the cement hydration reaction but also establish a robust interfacial bonding structure with the hydration products, consequently elevating the cement hydration efficiency [19,35].

The data presented in Fig 5(c) indicate that, in the concrete system modified with 6% CLDHs, a 10–30% increase in DS and RA dosages induces a 11.38% rise in the 3 d compressive strength and a 10.17% rise in the 28 d strength. Although the 28 d strength of this blend is 1.90% inferior to the control group, it still fulfills the performance standards of C50 concrete. This performance optimization is attributed to the multi-dimensional cooperative effects of DS and RA. Physically, the binary particles of DS and RA form a gradient packing structure, which not only fills the inter-aggregate gaps but also refines the pore structure of the matrix. The rigid skeleton formed by these particles facilitates uniform stress transfer and suppresses local cracking. Chemically, the active SiO_2_ in DS is activated in the alkaline environment, exerting a pozzolanic effect. The ultrafine particles supply numerous nucleation sites to accelerate hydration and react with CH to generate a stable C-S-H gel structure. At the interfacial transition zone, the unhydrated residues on RA surface synergize with active components of DS to form a dense interfacial structure, which optimizes crystal arrangement and strengthens interfacial adhesion. Collectively, the synergy of physical densification and chemical reaction culminates in the enhancement of concrete compressive strength [15,36].

### 3.2 The influence of DS, RA and CLDHs on the electrical flux of DSRAC

The chloride ion diffusion coefficient is a key parameter characterizing the diffusion characteristics of dissolved free ions in pore water under fully saturated conditions. The specimens in this test were vacuum saturated to a fully saturated state. In this paper, the empirical formula for converting chloride ion electrical flux to diffusion coefficient proposed by Feng et al. [37] was adopted to calculate the chloride ion diffusion coefficient, as shown in Eq. (5).


$$\begin{array}{c} D_{Cl}=\text{2.57765\textasciitilde{}+\textasciitilde{}0.00492Q} \end{array}$$ (5)

In the formula, *D*_*Cl*_ is the diffusion coefficient of Cl^-^ (×10^−9^ cm^2^/s), and *Q* is the total charge conducted in 6h (C).

**Fig 6:**
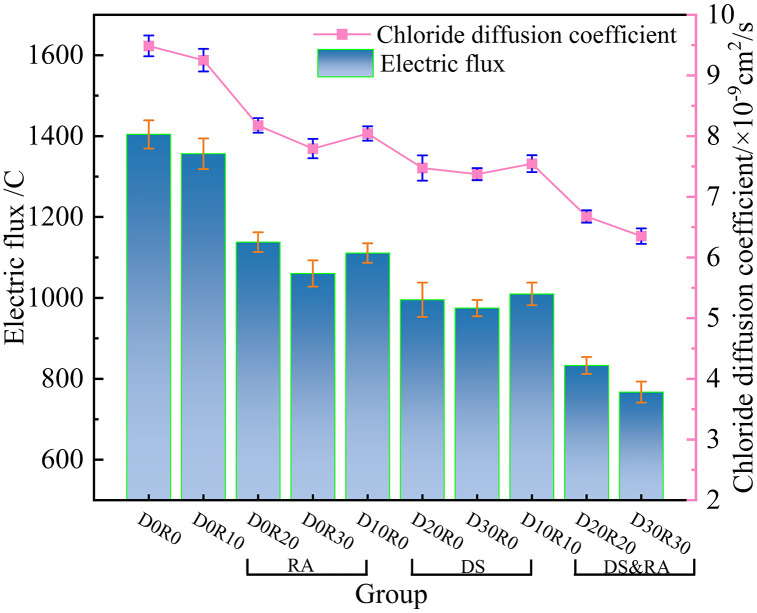
Changes of electric flux and chloride diffusion coefficient.

Fig 6 illustrates the evolution of electric flux and chloride ion diffusion coefficient for four distinct DSRAC mixtures at 28 d of curing. The experimental findings demonstrate that both chloride penetration resistance indicators exhibit a significant negative correlation with the dosages of CLDHs, DS, and RA. With the incorporation of CLDHs, the electric flux values of the RA group, DS group, and the combined DS-RA replacement group are all lower than that of the control group. Notably, in the DSRAC system, when the CLDHs content is 6% and both DS and RA replacement rates reach 30%, the electric flux and chloride ion diffusion coefficient achieve the lowest values across all test groups. Relative to the control group, this mixture shows a 45.37% reduction in electric flux, representing a 27.66% decrease compared to the D0R30 group and a 21.30% decrease compared to the D30R0 group. The results from single-dosage systems reveal that the electric flux in the RA group declines as the recycled aggregate replacement rate rises: increasing the RA replacement level from 10% to 30% results in a 21.82% reduction in electric flux. Similarly, the electric flux in the DS group decreases with increasing DS replacement rate, with a 12.27% reduction observed when the DS content rises from 10% to 30%. In the DSRAC compound-dosage system, the electric flux drops significantly with the concurrent increase in both RA and DS replacement rates, yielding a 24.04% reduction when both rates are elevated from 10% to 30%.

**Fig 7:**
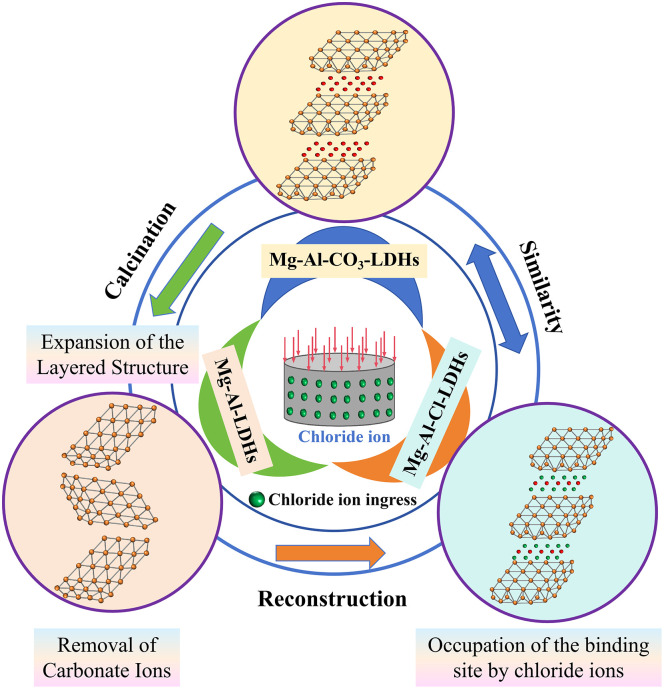
Schematic diagram of CLDHs material adsorption of chloride ions.

The reduction of DSRAC’s electrical flux and chloride ion diffusion coefficient is attributed to the coupled effect of CLDHs, DS and RA in multi-component coordination of microstructure and blocking of ion transport pathways. Physically, DS, as small-sized aggregates randomly distributed, reduces the connectivity of capillary pores and forms closed pores, directly blocking the penetration of chloride ions. Its fine particles and RA form a complementary particle size gradient gradation, and with the increase of the substitution rate, the filling effect is enhanced, reducing the matrix porosity and prolonging the complex ion migration path. The combined effect of the two is superior to that of RC and DS single addition systems [38]. Chemically, CLDHs, relying on the interlayer anion exchange property, fix chloride ions through physical adsorption and chemical curing, while refining the crystal grains of hydration products and regulating the pore solution environment, ensuring the full hydration and enhancing the matrix compactness [39]. Fig 7 shows a schematic diagram of CLDHs adsorbing chloride ions. At the interface level, the active SiO_2_ in DS triggers the pozzolanic effect, and the cement hydration product CH reacts to form dense C-S-H gel, filling the defects in the ITZ; the unhydrated particles on the surface of RA participate in secondary hydration, and together with DS, optimize the ITZ structure and enhance the interfacial bonding force, blocking the interface penetration path [31]. The coupling effect of physical filling, chemical curing and pozzolanic reaction significantly improves the chloride ion penetration resistance of DSRAC, and the higher the dosage of CLDHs, DS and RA, the more prominent the synergistic effect, making the two indicators significantly negatively correlated with the dosage of the three.

### 3.3 Pore structure analysis

NMR technology is an efficient and non-destructive testing method for characterizing the internal pore structure features of concrete-based materials. Its detection principle relies on the signal response of bound water in the pores within the concrete. By performing inversion analysis on the detection data, the evolution law of the transverse relaxation spectrum of the sample to be tested can be obtained.

**Fig 8:**
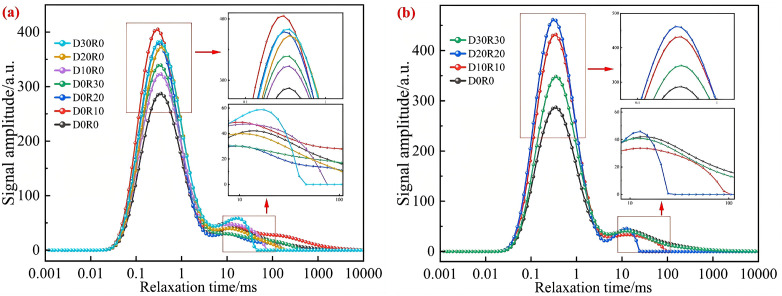
T_2_ spectrum. **(a)** RA, DS, **(b)** RA&DS.

As shown in Fig 8, the T_2_ relaxation spectrum of DSRAC presents two distinct characteristic peaks, with the first peak being the dominant one. Since the T_2_ relaxation time is closely related to the binding degree and mobility of hydrogen protons in DSRAC, this parameter can be used to quantitatively characterize the size and distribution of internal pores in the material [40,41]. The amplitude of the T_2_ spectrum is positively correlated with the number of pores within the corresponding pore diameter range – the higher the amplitude, the greater the number of pores in that specific size range. Notably, the main peak of DSRAC is concentrated in the T_2_ relaxation time range of 0.1 to 1 ms, showing the characteristics of short relaxation time and high peak intensity. This phenomenon indicates that the small pores corresponding to this relaxation time have the largest proportion in DSRAC and are the main component of the pore system; the second peak appears in the relaxation time range of 10–100 ms, with an extended relaxation time and significantly reduced peak intensity, reflecting that the volume fraction of medium and large pores in DSRAC is relatively small. Additionally, the intensity of the first peak in all DSRAC groups is generally higher than that of the blank control group, which confirms that the composite incorporation of RA, DS, and CLDHs has a significant regulatory effect on the pore structure characteristics, making the pore structure mainly concentrated in the radius range of 0.01 to 1 μm.

**Fig 9:**
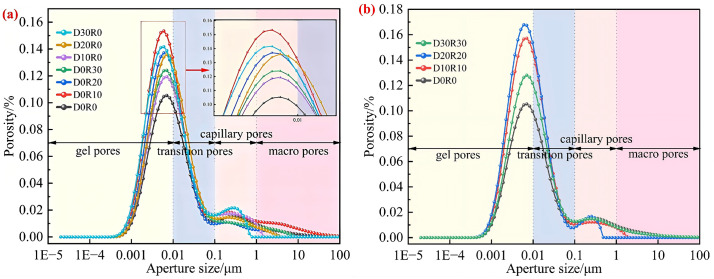
Influence of pore size distribution. **(a)** RA, DS, **(b)** RA&DS.

Fig 9 shows the influence of different ratios on the pore size distribution of DSRAC. According to the classification standard of concrete pore structure, the pores are divided into four intervals: gel pores (<0.01 μm), transition pores (0.01 ~ 0.1 μm), capillary pores (0.1 ~ 1 μm), and large pores (>1 μm). The main peaks of all groups are concentrated in the 0.1 ~ 1 μm interval, but the overall pore proportion shows a pattern of “gel pores> transition pores> capillary pores> large pores”, indicating that the matrix compactness of DSRAC is fundamentally good.

In the series where the dosage of CLDHs is 1% and the RA replacement rate is 10% to 30%, the main peak intensity of the capillary pore interval in the D0R0 blank control group is the lowest, corresponding to the least number of pores. In the D0R10, D0R20, and D0R30 groups, as the RA replacement rate gradually increases from 10% to 30%, the main peak intensity of the gel pore interval shows a continuous upward trend, and the peak width slightly widens. This phenomenon indicates that the incorporation of RA significantly increases the number of capillary pores inside the concrete and leads to a more dispersed pore distribution. The core reason for this is that the surface roughness of RA is relatively high and its water absorption rate is significantly higher than that of natural aggregates, which easily forms additional interface pores in the ITZ, thereby deteriorating the uniformity of the pore structure.

In the single DS blending series D10R0, D20R0, and D30R0 with a CLDHs dosage of 3%, as the DS substitution rate increases from 10% to 30%, the intensity of the main peak in the capillary pore range shows a gradually decreasing trend, confirming that DS, with its fine particle size distribution advantage, can effectively reduce the number of capillary pores through a physical filling effect and optimize the pore system. However, in the composite blending series D10R10, D20R20, and D30R30 with a CLDHs dosage of 6% and a synchronous increase in the substitution rates of DS and RA, the intensity of the main peak of capillary pores shows a “first decrease then increase” characteristic, and the peak position shifts towards smaller pore diameters. The DS-RA composite blending at low to medium substitution rates (10% to 20%) can synergistically optimize the pore structure, while at a high substitution rate of 30%, the interface defect effect of RA gradually becomes prominent, partially offsetting the filling optimization effect of DS. Additionally, comparing the overall peak intensity characteristics of CLDHs dosages of 1% and 3% in Fig 9(a) with those of 6% in Fig 9(b), the intensity of the main peak of capillary pores in all groups in Fig 9(b) is generally lower, indicating a synergistic effect of the combined addition of the three: CLDHs optimizes the interface transition zone through pozzolanic reaction, DS exerts a physical filling effect, and the negative pore effect of RA is significantly suppressed when added in appropriate amounts. Among them, the D30R30 group shows the best pore structure characteristics, suggesting that with an appropriate high dosage combination, the resource utilization of RA and DS can be achieved, while significantly enhancing the micro-density of concrete.

**Fig 10:**
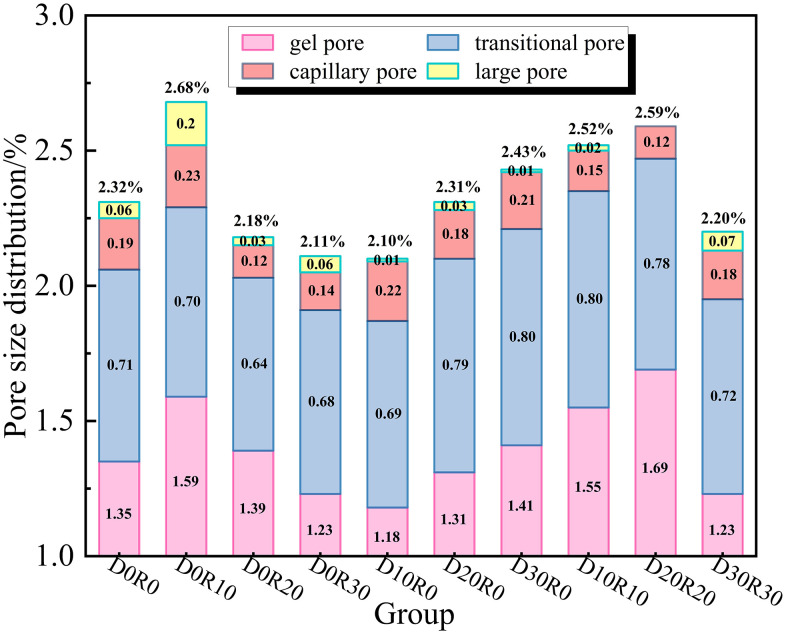
The distribution of the proportions of various types of pores in DSRAC.

Fig 10 shows the distribution characteristics of various pore ratios of DSRAC under different proportions. It can be seen from the figure that when the CLDHs dosage is 1%, the total porosity of DSRAC decreases with the increase of RA replacement rate. When the RA replacement rate is 30%, the total porosity is 2.11%, among which the gel pores, transition pores, capillary pores and macropores account for 1.23%, 0.68%, 0.14% and 0.06% respectively. The total porosity under this proportion is 9.05% lower than that of the blank control group. When the CLDHs dosage is 3%, the total porosity increases with the increase of DS replacement rate. When the DS replacement rate is 30%, the total porosity reaches 2.43%, and the corresponding gel pores, transition pores, capillary pores and macropores account for 1.41%, 0.80%, 0.21% and 0.01% respectively. The total porosity is 4.74% higher than that of the blank control group. When the CLDHs dosage is 6%, the total porosity first increases and then decreases with the increase of DS and RA replacement rates. When both replacement rates are 30%, the total porosity is 2.20%, and the pore ratios are gel pores 1.23%, transition pores 0.72%, capillary pores 0.18% and macropores 0.07% respectively. The total porosity is 5.17% lower than that of the blank control group. Overall, the distribution of various types of pores in DSRAC shows a pattern of gel pores > transition pores > capillary pores > macropores. When the CLDHs dosage is 1%, the RA undergoes secondary hydration under the induction of crystal nuclei, generating C-S-H gel to fill the internal pores, so the total porosity decreases with the increase of RA replacement rate. When the CLDHs dosage is 3%, the DS basically has no hydration activity. At high replacement rates, the fine particle gradation is single and the accumulation defects increase, resulting in an increase in total porosity with the increase of DS replacement rate. The two have different action mechanisms, so they show opposite trends. It should be noted that the determination of “optimal comprehensive performance” in this study is not based solely on the single indicator of total porosity, but takes into account multiple performance dimensions such as compressive strength, chloride ion diffusion coefficient, and pore structure distribution. Although the total porosity of the D30R30 group is slightly higher than that of the D0R30 group, it has a higher gel pore ratio and a better transition pore ratio, and performs well in compressive strength and chloride ion penetration resistance. At the same time, it realizes the high-value utilization of DS and RA, so it is comprehensively determined as the optimal proportion.

### 3.4 SEM analyze

**Fig 11:**
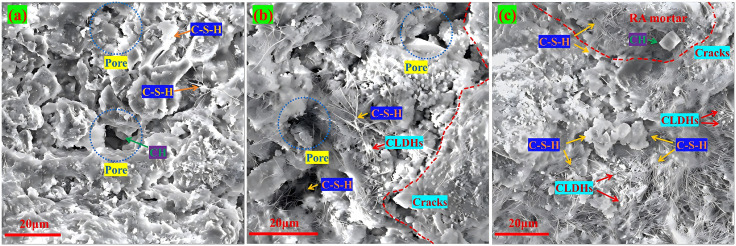
SEM images of the blank control group and RA concrete. **(a)** D0R0, **(b)** D0R10, **(c)** D0R30.

Fig 11 shows the SEM micrographs of the blank control group and recycled aggregate concrete. Among them, Fig 11(a) is the SEM image of the blank control group. It can be seen from the figure that the flocculent C-S-H gel is evenly distributed in the system and interwoven to form a complete skeleton of the concrete matrix. The pore type is mainly gel pores, and no obvious microcracks are observed in the microstructure, with good overall integrity. In terms of macroscopic mechanical properties, this dense microstructure provides a good carrier for stress transmission and effectively avoids stress concentration. However, due to the absence of CLDHs, the nucleation induction effect is lacking, the cement hydration reaction is not sufficient, and the amount of hydration products generated is limited, which restricts the strength growth. At the same time, there are many un-filled connected pores in the microstructure, and the ITZ is relatively loose and wide. Under the action of external force or internal stress, it is easy to generate microcracks, which not only weakens the further improvement space of mechanical properties but also keeps the chloride ion diffusion coefficient at a high level. This microstructure feature is consistent with the macroscopic performance test results, fully demonstrating the dominant role of microstructure on macroscopic performance.

As can be seen from Fig 11(b), when the CLDHs dosage is 1%, it mainly plays a role in inducing crystal nuclei and, in conjunction with the secondary hydration of RA, jointly regulates the performance. In the D0R10 group with a 10% RA replacement rate, the amount of surface-hardened mortar is small, the secondary hydration is insufficient, and interface transition zone defects are introduced. As shown in Fig 11(b), there are pores and small cracks, resulting in a decrease in the 28 d compressive strength compared to the blank control group, and a slight decrease in the chloride ion diffusion coefficient. In Fig 11(c), in the D0R30 group with a 30% RA replacement rate, there is a considerable amount of surface-hardened mortar that can undergo secondary hydration reactions, generating a large amount of C-S-H gel, which fills the pores and heals the interface cracks. As shown in Fig 11(c), the pores and cracks are filled with hydration products, and the pore connectivity is reduced, demonstrating the synergistic optimization effect of the two.

**Fig 12:**
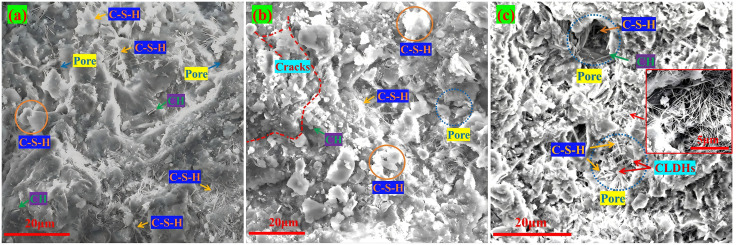
SEM images of DS concrete. **(a)** D10R0, **(b)** D20R10, **(c)** D30R0.

Fig 12 shows the SEM images of desert sand concrete. When the CLDHs dosage is 3%, DS is the variable for single replacement. The physical filling effect of DS and the hydration induction and crack inhibition effect of CLDHs are superimposed, affecting the performance evolution and crack development. As the DS replacement rate increases from 10% to 30%, the physical filling effect of DS fine particles is enhanced, filling the tiny voids between the cement matrix and aggregates. Meanwhile, CLDHs continuously induce cement hydration, as shown in Fig 12(c), the C-S-H gel increases, and the hydration product network becomes denser. Moreover, the layered structure of CLDHs adsorbs free ions, reduces interfacial stress, and inhibits crack initiation and propagation. At low replacement rates, no obvious cracks are observed, while at high replacement rates, only a few disconnected tiny cracks exist. However, the desert sand has a relatively fine particle size and a single gradation. At high replacement rates, the excessive fine particles reduce the packing density of aggregates, resulting in more voids that cannot be filled, as shown in Fig 12(b) and 12(c). The uneven particle packing causes local stress concentration and induces tiny cracks. These defects limit the hydration filling and crack inhibition effects, which is consistent with the macroscopic result of a gentle decrease in the chloride ion diffusion coefficient mentioned earlier. The chloride ion diffusion coefficient decreases from 8.04 × 10^−9^ cm²/s to 7.37 × 10^−9^ cm²/s, with a gentle decline, and cannot be improved simultaneously with the strength.

**Fig 13:**
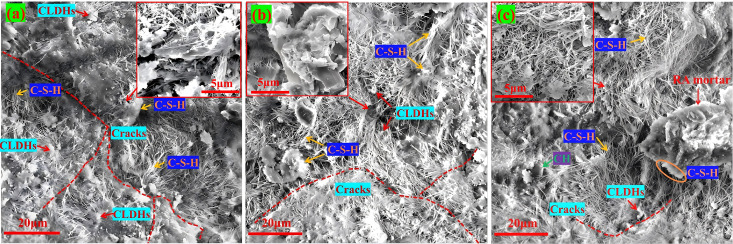
SEM images of RA&DS concrete. **(a)** D10R10, **(b)** D20R20, **(c)** D30R30.

Fig 13 shows the SEM images of RA and DS concrete. When the CLDHs dosage is 6%, the mechanism of action changes to a dual effect of crystal nucleus induction and ion exchange, which has both crack inhibition and healing capabilities, and works in synergy with DS and RA to achieve the best regulatory effect. In the D10R10 group, when the replacement rates of DS and RA are both 10%, the particle gradation is not optimal, and the uneven accumulation of aggregates causes stress concentration. The synergistic hydration effect of the two has not been fully exerted, and the hydration products are insufficient, unable to effectively fill the pores and inhibit cracks. As shown in Fig 13(a), the hydration filling is insufficient, and there are connected cracks, with a generally moderate micro-density. In the D20R20 group, when the replacement rates of both are 20%, a preliminary multi-level filling system is formed, increasing the density of aggregate accumulation, alleviating stress concentration, and reducing crack initiation. The ion exchange of CLDHs optimizes the pore solution environment, promoting thorough hydration. As shown in Fig 13(b), there is an increase in C-S-H gel, a more complete hydration network, filling pores and healing some minor cracks, but there are still pores and obvious connected cracks. In the D30R30 group, when the replacement rates of both are 30%, the synergy effect is the best. The ITZ is significantly densified and its width is significantly reduced. No obvious connected cracks are observed, only a few scattered small gel pores exist, forming an ideal multi-level filling system, reducing the voids in aggregate accumulation, avoiding stress concentration, and inhibiting crack initiation. The high dosage of CLDHs’ dual effect and the secondary hydration of recycled aggregates promote each other, generating a large amount of dense hydration products, forming a continuous and complete network, filling transition pores and some large pores, and healing minor cracks. Although the total porosity of 2.20% is slightly higher than that of the D0R30 group, the hydration products preferentially fill the connected transition pores and cracks, blocking the chloride ion penetration channels. The 28 d compressive strength reaches 53.1 MPa, close to the blank group, and the chloride ion diffusion coefficient is reduced to the lowest 6.35 × 10^−9^ cm²/s, achieving simultaneous optimization of mechanical and durability properties.

In summary, the macroscopic performance of DSRAC is closely related to its microstructure and crack development. The core mechanism for microstructure optimization and macroscopic performance improvement is composed of CLDHs regulation, RA secondary hydration, and DS multi-level filling. CLDHs promote hydration through nucleation effect and ionic curing, refine the pore structure and densify the ITZ, which directly corresponds to the refinement of the pore structure and the improvement of macroscopic performance. The characteristics of hydration products determine the state of pores and cracks, and the pores and cracks jointly affect the macroscopic performance. The joint optimization of the two is the key to performance improvement.

### 3.5 XRD analyze

**Fig 14:**
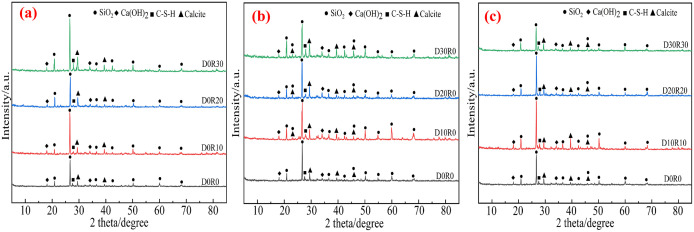
Different types of DSRAC’s XRD patterns. **(a)** RA, **(b)** DS, **(c)** DS&RA.

Fig 14 shows the XRD patterns of different types of DSRAC. By combining XRD phase characterization with SEM microscopic morphology observation, the intrinsic mechanism of CLDHs and RA and DS synergistically regulating the microstructure evolution and macroscopic performance of concrete can be clearly revealed. In this paper, the evolution law of hydration products is visually reflected by semi-quantitative comparison of characteristic peak intensities. The peak intensity of Ca(OH)2 (2θ ≈ 18°) is used to reflect the consumption degree of CH, and the peak intensity and broadening degree of C-S-H gel (2θ ≈ 29°) are used to reflect the generation amount of gel.

In Fig 14(a), the blank control group shows a relatively high intensity of the Ca(OH)_2_ characteristic peak and a gentle C-S-H gel diffraction peak, which is highly consistent with the microscopic characteristics of insufficient hydration reaction and numerous interconnected pores observed by SEM. The enrichment of Ca(OH)_2_ indicates that the system lacks crystal nucleus sites to induce the formation of C-S-H gel, revealing from a microscopic perspective the reasons for the limited macroscopic strength growth and the relatively high chloride ion diffusion coefficient. In the D0R10 group, the Ca(OH)_2_ peak slightly decreases and the C-S-H peak slightly increases, but the phase evolution is limited, corresponding to the phenomenon of less hardened mortar on the RA surface and insufficient secondary hydration observed by SEM. The nucleation induction effect of CLDHs fails to counteract the interface defects introduced by RA, resulting in a decrease in 28 d compressive strength and only a slight reduction in the chloride ion diffusion coefficient. In the D0R30 group, the Ca(OH)_2_ peak significantly weakens, and the C-S-H gel diffraction peak broadens and intensifies, which is consistent with the morphology observed by SEM where pores and cracks are filled with hydration products; the abundant active components provided by 30% RA undergo sufficient secondary hydration under the induction of CLDHs, generating a large amount of C-S-H gel to fill the defects, achieving an improvement in mechanical properties and a significant decrease in the chloride ion diffusion coefficient.

In Fig 14(b), as the DS substitution rate increases from 10% to 30%, the characteristic peak of Ca(OH)_2_ continuously weakens, while the diffraction peak of C-S-H gel gradually broadens and its intensity increases, which is consistent with the SEM observation of “increased C-S-H gel and denser hydration network”, indicating that the CLDHs nuclei continuously activate the cement hydration and, in combination with the physical filling effect of DS fine particles, optimize the microstructure of the matrix. No new characteristic diffraction peaks appeared in the XRD, confirming that CLDHs mainly adsorb free ions through their layered structure and relieve interface stress, supporting the SEM observation that there are no obvious cracks at low substitution rates and only a few disconnected microcracks at high substitution rates. However, when the DS substitution rate reaches 30%, the enhancement amplitude of the C-S-H peak and the decline rate of the Ca(OH)_2_ peak both slow down, verifying the SEM observation that “excessive fine particles reduce the packing density of aggregates and create unfilled voids”, reflecting the stress concentration caused by the single gradation of DS at high substitution rates, which limits the hydration filling effect and explains the phenomenon that the chloride ion diffusion coefficient and strength do not increase simultaneously.

In Fig 14(c), the D10R10 group shows a relatively high intensity of the Ca(OH)_2_ peak and a gentle C-S-H peak, corresponding to the SEM features of insufficient hydration products and numerous interconnected cracks. This is attributed to the inactive induction effect of CLDHs and poor particle gradation causing stress concentration. The D20R20 group exhibits a weakened Ca(OH)_2_ peak and a broadened and enhanced C-S-H peak, corresponding to a well-developed hydration network in the SEM. The ion exchange effect of CLDHs and multi-level particle filling alleviate stress concentration, but the hydration is not fully completed. The D30R30 group shows a significant reduction in the peak of active SiO_2_ in DS and a substantial weakening of the Ca(OH)_2_ peak, with the C-S-H peak having the highest intensity. This corresponds to the microscopic characteristics of dense hydration products and densified ITZ in the SEM. The pozzolanic reaction of active SiO_2_ in DS and the secondary hydration of RA occur fully, combined with the dual effects of CLDHs, achieving simultaneous optimization of macroscopic performance.

### 3.6 Quantitative relationship analysis of macroscopic performance and pore structure of DSRAC based on grey prediction modell

#### 3.6.1 Grey entropy correlation analysis.

Grey entropy correlation analysis is a refined optimization of the classical grey correlation analysis framework, which allows for the consistent quantification of multiple influencing factors to delineate their respective degrees of influence on evaluation indices. The method is characterized by two primary strengths: it requires only a small sample dataset and mitigates the risk of over-amplifying the weight of any single factor. Owing to these advantages, it has been extensively implemented in a wide spectrum of research and industrial applications [42–43].

To reveal the intrinsic connection between the macroscopic performance and microstructure of DSRAC under the synergistic effect of CLDHs, DS and RA, this study adopts the grey entropy correlation analysis method to derive the grey entropy correlation degree between compressive strength and pore structure parameters. Given that the pore structure is the core component of the mesoscopic system of concrete, its complex characteristics cannot be comprehensively characterized solely by porosity, but are also regulated by multiple factors such as pore size scale and pore size distribution. Therefore, this study selects pore characteristic indicators and conducts grey entropy correlation analysis with the compressive strength and chloride ion diffusion coefficient of DSRAC respectively, with the aim of establishing a quantitative correlation model between pore structure characteristic parameters and the macroscopic performance of DSRAC. The specific process is as follows:

(1) Identify the initial sequence.

Within the framework of grey entropy correlation analysis, the reference sequence represents the target evaluation metric under the influence of multiple factors, and the comparison sequence corresponds to the primary influencing factors that exert an effect on the reference sequence. In the present study, the compressive strength and chloride ion diffusion coefficient of DSRAC were selected as the reference sequences, with total porosity, gel pore porosity, transition pore porosity, capillary pore porosity, macropore porosity, and other pore structure parameters employed as the comparison sequences.

(2) Dimensionless normalization.

The original experimental data were subjected to dimensionless normalization and mean normalization processing, as expressed in Eq. (6).


$$\begin{array}{c} {x^{\prime }}_{i}=\frac{x_{i}\left(k\right)}{\frac{\text{1}}{m}\sum_{i=1}^{m}x_{i}\left(k\right)}x_{i} \end{array}$$ (6)

In the formula, k=1,2,⋯,n; i=1,2,⋯,m.

(3) Computing the element-wise discrepancies between the reference and comparison sequences, as defined in Eq. (7).

$$\begin{array}{c} \Delta_{i}\left(k\right)=\left|y\left(k\right)-x_{i}\left(k\right)\right| \end{array}$$ (7)

(4) Calculation of the largest and smallest disparities observed among all sequence pairs, as defined in Eq. (8).

$$\begin{array}{c} M=\text{max}\Delta_{i}(k),\;m=\text{min}\Delta_{i}(k) \end{array}$$ (8)

(5) Calculation of the correlation coefficients is as expressed in Eq. (9).

$$\begin{array}{c} \gamma_{i}\left(k\right)=\frac{m+\delta M}{\Delta_{i}\left(k\right)+\delta M} \end{array}$$ (9)

Within the equation, δ denotes the resolution parameter, typically assigned a value between 0.5 and 0.6; for this investigation, a value of 0.5 was adopted.

(6) The grey entropy correlation degree is computed using the formula presented in Eq. (10).

$$\begin{array}{c} R_{i}=\frac{\text{1}}{Q}\sum_{k=1}^{Q}\gamma_{i}\left(k\right) \end{array}$$ (10)

In the formula, k=1,2,⋯,n. The closer the grey entropy correlation degree is to 1, the stronger the correlation between each influencing factor and the reference sequence.

**Fig 15:**
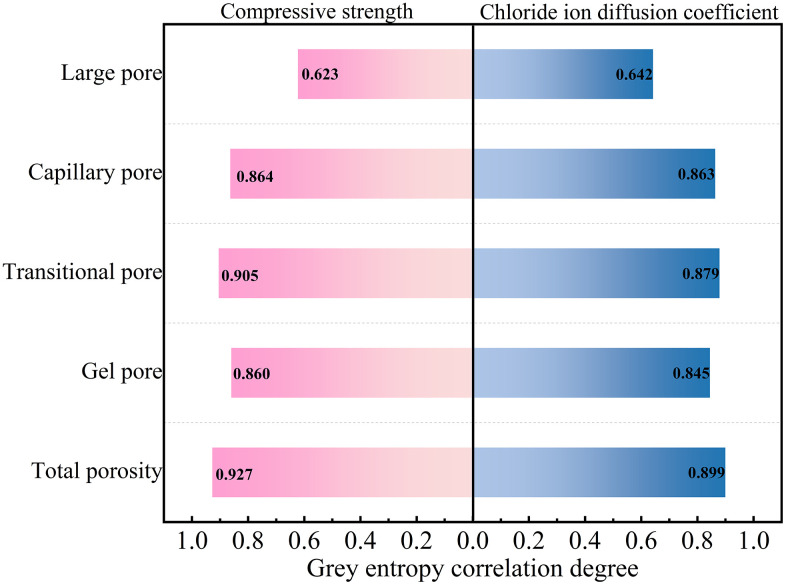
Correlation between macroscopic properties and gray entropy of pore parameters.

The outcomes of the grey entropy correlation analysis are presented in Fig 15. As demonstrated in the plot, the correlation magnitudes between the pore structure parameters and the compressive strength and chloride ion diffusion coefficient of DSRAC, ranked from highest to lowest, are as follows: total porosity > transition porosity > capillary porosity > gel porosity > macroporosity. The standard classification for correlation strength is defined as: a correlation coefficient ≥ 0.8 represents a strong correlation, 0.6 ≤ coefficient < 0.8 represents a moderate correlation, and a coefficient < 0.6 represents a weak correlation [44]. The correlation degrees of total porosity, transition porosity, capillary porosity, and gel porosity with both performance metrics of DSRAC are all above 0.8, signifying a strong correlation. Conversely, the correlation degrees of macroporosity with compressive strength and chloride ion diffusion coefficient are 0.623 and 0.624, respectively, indicating a moderate correlation.

#### 3.6.2 Establishment of the GM(1, 4) model.

The grey model has demonstrated significant advantages in the field of concrete macroscopic performance analysis, including strong feasibility, wide applicability, and excellent result accuracy. It can effectively address the challenges of limited experimental sample size and complex coupling of influencing factors in this field, providing a reliable theoretical tool for the quantitative analysis of the relationship between the macroscopic performance of DSRAC and its pore structure. Its application effectiveness has been verified in numerous studies [25,45]. The intrinsic connection between the macroscopic properties of DSRAC, such as compressive strength and chloride ion diffusion coefficient, and pore parameters is dominated by the gradual evolution process of the internal pore structure. This characteristic is highly consistent with the core advantage of the grey model in handling incomplete information systems, thus enabling effective research on the relationship between the compressive strength, chloride ion diffusion coefficient of DSRAC and its pore structure.

Based on the results of grey entropy correlation analysis, the pore structure parameters with strong correlation (correlation degree ≥ 0.8) to the macroscopic performance of DSRAC were selected as the model input variables. Among them, the gel pore porosity was a strongly correlated parameter and thus included in the model; the macropore porosity was only moderately correlated, so it was excluded. The modeling process strictly followed the strength of the correlation degree as the parameter screening basis to ensure the statistical rigor and prediction accuracy of the model, and GM(1,4) prediction models for compressive strength and chloride ion diffusion coefficient were respectively constructed. This model is applicable to the DSRAC system with CLDHs dosage of 0%–6% and DS and RA substitution rates of 0%–30%, and the modeling data were all based on the test results after standard curing for 28 d. The specific modeling process is as follows:

(1) Define the original data sequence $\overset{\left(0\right)}{\underset{i}{X}}$as shown in Eq. (11).

$$\begin{array}{c} \overset{\left(0\right)}{\underset{i}{X}}=\left(\overset{\left(0\right)}{\underset{i}{x}}\left(1\right),\overset{\left(0\right)}{\underset{i}{x}}\left(2\right),\text{\textbackslash{}ldots},\overset{\left(0\right)}{\underset{i}{x}}\left(n\right)\right) \end{array}$$ (11)

(2) The first-order cumulative generation operation is performed on the original sequence $\overset{\left(0\right)}{\underset{i}{X}}$to obtain the first-level cumulative sequence $\overset{\left(1\right)}{\underset{i}{X}}$, as shown in Eq. (12).

$$\begin{array}{c} \overset{\left(1\right)}{\underset{i}{X}}=\left(\overset{\left(1\right)}{\underset{i}{x}}\left(1\right),\overset{\left(1\right)}{\underset{i}{x}}\left(2\right),\text{\textbackslash{}ldots},\overset{\left(1\right)}{\underset{i}{x}}\left(n\right)\right) \end{array}$$ (12)

In the formula, The $\overset{\left(1\right)}{\underset{i}{x}}\left(n\right)=\sum_{i=1}^{n}\overset{\left(0\right)}{\underset{i}{x}}\left(i\right)$ sequence is close to the mean.。

(3) Construct the sequence of generation values adjacent to the mean, as shown in Eq. (13).

$$\begin{array}{c} \overset{\left(1\right)}{\underset{\text{1}}{Z}}=\left(\overset{\left(1\right)}{\underset{\text{1}}{z}}\left(1\right),\overset{\left(1\right)}{\underset{\text{1}}{z}}\left(2\right),\text{\textbackslash{}ldots},\overset{\left(1\right)}{\underset{\text{1}}{z}}\left(n\right)\right) \end{array}$$ (13)

In the formula, $\overset{\left(1\right)}{\underset{\text{1}}{z}}\left(n\right)=\left(\overset{\left(1\right)}{\underset{\text{1}}{x}}\left(n-1\right)+\overset{\left(1\right)}{\underset{\text{1}}{x}}\left(n\right)\right)/2$.

(4) In accordance with the sequence definitions provided above, $\overset{\left(0\right)}{\underset{\text{1}}{X}}$ is designated as either the compressive strength or chloride ion diffusion coefficient of DSRAC, while $\overset{\left(0\right)}{\underset{j}{X}}$ (j=2,3,4) denotes the selected pore structure parameters. Building on the preceding analysis, the GM (1,4) model is formulated herein, with its mathematical expression presented in Eq. (14).

$$\begin{array}{c} \overset{\left(0\right)}{\underset{\text{1}}{x}}\left(k\right)+p\overset{\left(1\right)}{\underset{\text{1}}{z}}\left(n\right)=\sum_{j=2}^{\text{4}}q_{j}\overset{\left(1\right)}{\underset{J}{x}}\left(k\right) \end{array}$$ (14)

In this equation, *p* denotes the development coefficient, which captures the overall evolutionary pattern of the sequence; meanwhile, q_2_, q_3_, and q_4_ serve as grey action quantities, quantifying how individual pore structural parameters contribute to the macroscopic mechanical behavior of DSRAC. *p* and the aforementioned *q*_*j*_ jointly form the correlation parameter matrix of the GM (1, 4) model, as shown in Eq. (15), thereby achieving the quantitative analysis of the intrinsic correlation among variables.


$$\begin{array}{c} C={\left[\begin{array}{cccc} p & q_{\text{2}} & q_{\text{3}} & q_{\text{4}} \end{array}\right]}^{T} \end{array}$$ (15)

(5) Parameter fitting is performed based on the least squares principle, and a vector equation for the coefficients of the predictive model is constructed, as presented in Eqs. (16) and (17).

$$\begin{array}{c} C={\left(A^{T}A\right)}^{-1}A^{T}B=\left[\begin{array}{cccc} p & q_{\text{2}} & q_{\text{3}} & q_{\text{4}} \end{array}\right] \end{array}$$ (16)

$$\begin{array}{c} A=\left[\begin{array}{cccc} -\overset{\left(1\right)}{\underset{\text{1}}{z}}\left(2\right) & \overset{\left(1\right)}{\underset{\text{2}}{x}}\left(2\right) & \cdots & \overset{\left(1\right)}{\underset{\text{4}}{x}}\left(2\right)\\ -\overset{\left(1\right)}{\underset{\text{1}}{z}}\left(2\right) & \overset{\left(1\right)}{\underset{\text{2}}{x}}\left(3\right) & \cdots & \overset{\left(1\right)}{\underset{\text{4}}{x}}\left(3\right)\\ \vdots & \vdots & \vdots & \vdots \\ -\overset{\left(1\right)}{\underset{\text{1}}{z}}\left(2\right) & \overset{\left(1\right)}{\underset{\text{2}}{x}}\left(n\right) & \cdots & \overset{\left(1\right)}{\underset{\text{4}}{x}}\left(n\right) \end{array}\right] \end{array}$$ (17)

Based on the DSRAC specimens prepared with different dosages of CLDHs, DS and RA replacement rates, taking their compressive strength and chloride ion diffusion coefficient as evaluation indicators, and four pore parameters (total porosity, transition pore porosity, capillary pore porosity, gel pore porosity) as modeling factors, the compressive strength model and chloride ion diffusion coefficient model of DSRAC were respectively established for the effect of different factors, and the expressions are shown in Eqs. (18) and (19).

DSRAC compressive strength model:


$$\begin{array}{c} \overset{\left(0\right)}{\underset{\text{1}}{x^{\prime }}}\left(n\right)=-0.210\overset{\left(1\right)}{\underset{\text{2}}{x}}\left(n\right)-18.467\overset{\left(1\right)}{\underset{\text{3}}{x}}\left(n\right)+62.351\overset{\left(1\right)}{\underset{\text{4}}{x}}\left(n\right)-73.515z^{\left(1\right)}\left(n\right) \end{array}$$ (18)

DSRAC chloride Ion diffusion coefficient model:


$$\begin{array}{c} \overset{\left(0\right)}{\underset{\text{1}}{x^{\prime }}}\left(n\right)=0.056\overset{\left(1\right)}{\underset{\text{2}}{x}}\left(n\right)-3.641\overset{\left(1\right)}{\underset{\text{3}}{x}}\left(n\right)+12.200\overset{\left(1\right)}{\underset{\text{4}}{x}}\left(n\right)-3.178z^{\left(1\right)}\left(n\right) \end{array}$$ (19)

Based on the grey entropy correlation analysis and the test results, this paper determines the key pore structure thresholds that affect the macroscopic performance of DSRAC: total porosity ≤ 2.20% and the proportion of transition pores ≥ 0.70%. Specimens that reach the threshold range have the best mechanical properties and chloride ion penetration resistance, which can provide direct references for material selection and mix proportion design in engineering.

#### 3.6.3 Model validation.

**Fig 16:**
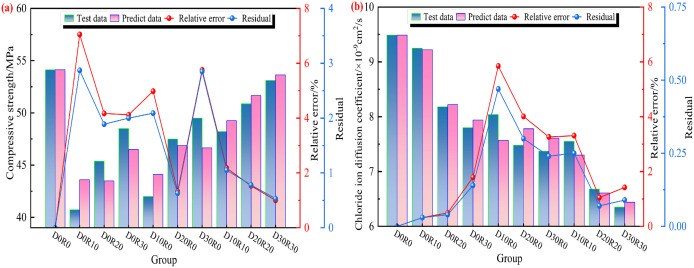
Test date and Predicted date of GM(1,4) model. **(a)** Compressive strength, **(b)** Chloride ion diffusion coefficient.

The comparison results of the predicted values of compressive strength and chloride ion diffusion coefficient of DSRAC based on the GM(1, 4) model and the experimental measured values are shown in Fig 16. As can be seen from Fig 16(a), the predicted values of compressive strength are in good agreement with the measured values, with the maximum residual being 2.85 MPa and the maximum relative error being 7.05%. From Fig 16(b), it can be seen that the predicted values of the chloride ion diffusion coefficient have a good fitting effect with the measured values, with the maximum residual being 0.47 × 10^−9^ cm^2^/s and the maximum relative error being 5.85%. Overall, the predicted values of the two performance indicators are highly consistent with the measured values, and the relative errors of the GM(1,4) model for these two indicators are both controlled within 8%.

The above results confirm that the constructed GM(1,4) model has high prediction accuracy and can effectively quantify the compressive strength and chloride ion diffusion coefficient of DSRAC based on the pore structure parameters selected by grey entropy correlation analysis, providing reliable theoretical support and technical basis for the performance evaluation of DSRAC.

## 4. Conclusions

In the present investigation, the dosage of CLDHs and the substitution ratios of DS and RA were selected as variables. A suite of analytical techniques, including NMR, SEM, XRD, compressive strength testing, and electric flux measurement, was employed to systematically explore the influence of multi-factor coupling on the compressive strength and chloride ion penetration resistance of DSRAC. On this foundation, the grey entropy correlation analysis method was utilized to quantitatively elucidate the correlation between the pore structure parameters of DSRAC and both compressive strength and chloride ion diffusion coefficient. Moreover, a high-precision GM (1,4) grey prediction model was constructed to reliably predict the compressive strength and chloride ion diffusion coefficient, with pore structure parameters as input variables. The main findings are outlined below:

(1) The co-addition of CLDHs, DS and RA can synergistically optimize the macroscopic performance of DSRAC. When the CLDHs dosage is 6% and the replacement rates of DS and RA are both 30%, the comprehensive performance of DSRAC is the best. The compressive strength only decreases by 1.9% compared with the blank control group, still meeting the C50 strength grade requirement. The chloride ion diffusion coefficient decreases by 33.09%, achieving a synergistic optimization of mechanical properties and durability.
(2) The pores of DSRAC are mainly gel pores, distributed in the order of “gel pores> transition pores> capillary pores> large pores”. The total porosity and the proportion of transition pores are the key parameters affecting the macroscopic performance. The variation law of total porosity is significantly different with different CLDHs dosages: it decreases with the increase of RA replacement rate at 1% dosage; it increases with the increase of DS replacement rate at 3% dosage; at 6% dosage, it first increases and then decreases with the increase of the combined replacement rate of DS and RA.
(3) The mechanism of multi-component synergistic regulation of the pore structure of DSRAC has obvious dosage dependence. At 1% CLDHs dosage, RA secondary hydration is activated, filling the pores and reducing the porosity; at 3% CLDHs dosage, the porosity increases due to the insufficient activity and single gradation of DS; at 6% CLDHs dosage, the porosity increases at low replacement rates, while at high replacement rates, it decreases through multi-level filling and synergistic hydration, achieving a denser structure.
(4) The grey entropy correlation analysis shows that the correlation strength between the pore structure parameters of DSRAC and its macroscopic performance is in the order of: total porosity > transition pore porosity > capillary pore porosity > gel pore porosity > large pore porosity, with the first four showing strong correlations. The GM (1,4) model constructed based on this has high prediction accuracy, with relative errors all below 8%, providing reliable theoretical support for the quantitative assessment and engineering application of the mechanical properties and chloride ion penetration resistance of DSRAC.

This study is based on the actual engineering construction needs in the desert and salinized areas of southern Xinjiang, focusing on the engineering problems such as the shortage of river sand resources, the low utilization rate of construction waste, and the susceptibility of concrete structures to chloride ion erosion. The DSRAC experimental research was carried out. Both desert sand and recycled aggregates are locally available materials, which can be sourced locally, significantly reducing the transportation cost of raw materials, and have both environmental and economic benefits. They can provide a stable local raw material supply for green concrete structure engineering in desert and salinized areas, and have good engineering promotion value and practical application prospects. The test results show that when the CLDHs dosage is 6% and the replacement rates of desert sand and recycled aggregates are both 30%, the comprehensive performance of DSRAC is the best. It can significantly improve the resistance to chloride ion penetration while ensuring mechanical properties, meeting the usage requirements of load-bearing structures in a chloride salt erosion environment.

This study still has certain limitations. The current tests only studied the material performance under standard curing conditions and did not consider the complex working conditions such as freeze-thaw cycles, dry-wet-salt erosion coupling, load action, and long-term service aging in actual engineering. In addition, this study used the grey theory model to predict the macroscopic performance based on the pore structure parameters, which has high accuracy, but the test data points and long-term age verification still need to be supplemented. Subsequent research can conduct long-term durability tests, component force performance studies, and on-site working condition simulations to further improve the data density of the model and enhance the engineering applicability and reliability of the research conclusions.

## Supporting information

S1 Fig Fig 3(a) shows the photo of natural aggregates after crushing is completed, and Fig 3(b) shows the photo of recycled aggregates after crushing is completed. (TIF)
