# Supplementary figures and images for: Research on pore characteristics and macroscopic performance quantitative prediction of desert sand recycled aggregate concrete based on NMR and grey theory
Source: PLoS One. 2026 Jun 3;21(6):e0349560. doi: 10.1371/journal.pone.0349560 (PMC13232806; doi:10.1371/journal.pone.0349560)

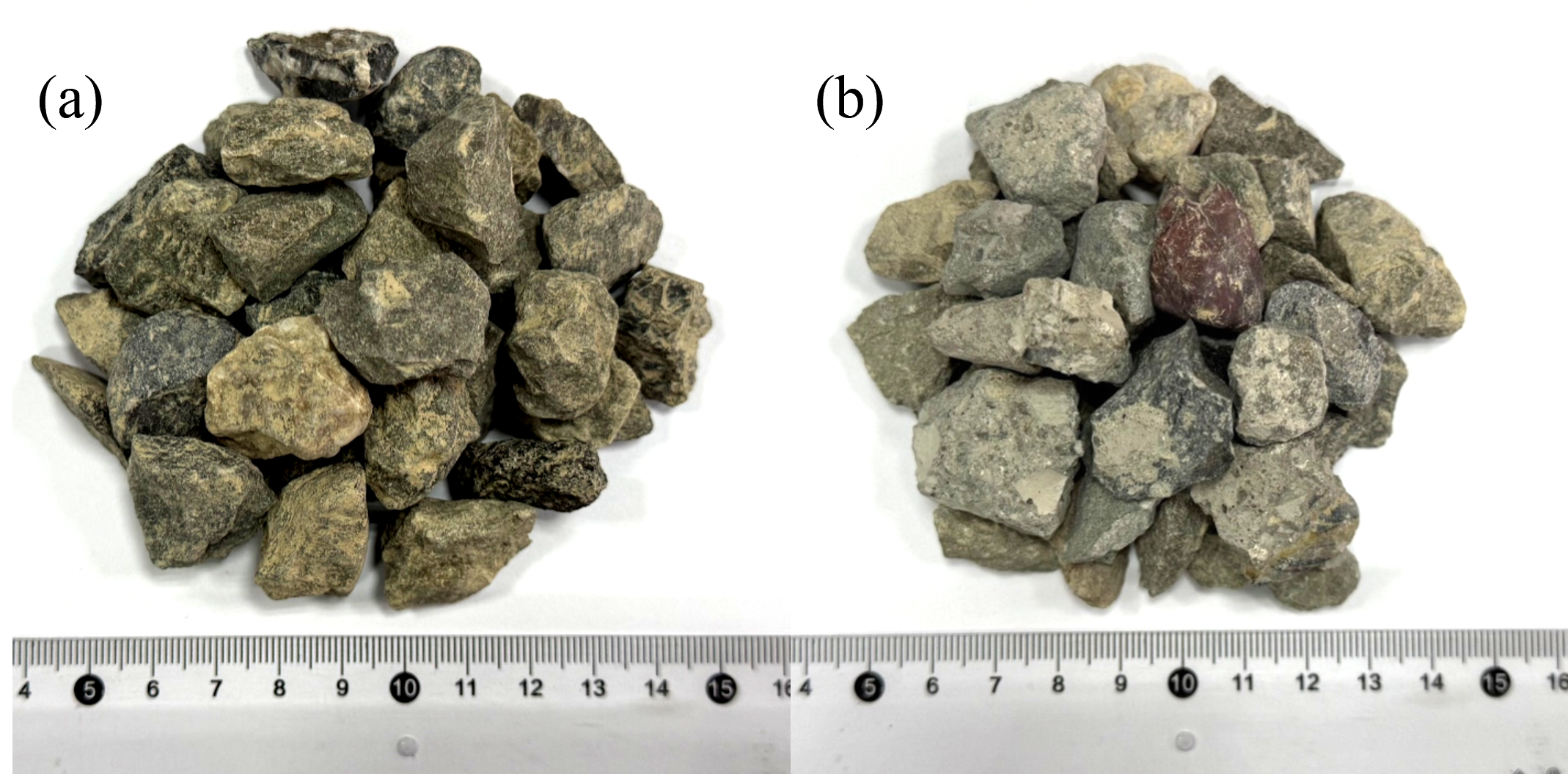

Supplement: S1 Fig — (TIF) [file pone.0349560.s001.tif]
